# CD9P-1 expression correlates with the metastatic status of lung cancer, and a truncated form of CD9P-1, GS-168AT2, inhibits *in vivo* tumour growth

**DOI:** 10.1038/sj.bjc.6606033

**Published:** 2011-01-04

**Authors:** W Guilmain, S Colin, E Legrand, J P Vannier, C Steverlynck, M Bongaerts, M Vasse, S Al-Mahmood

**Affiliations:** 1 Gene Signal Research Center, 4 Rue Pierre Fontaine, Evry 91000, France; 2 Groupe de recherche MERCI (EA 3829), Faculté de Médecine & Pharmacie, Rouen 76000, France

**Keywords:** CD9P-1, CD9, lung cancer, tetraspanin, tumour growth

## Abstract

**Background:**

Loss of CD9 expression has been correlated with a higher motility and metastatic potential of tumour cells originating from different organs. However, the mechanism underlying this loss is not yet understood.

**Methods:**

We produced a truncated form of partner 1 of CD9 (CD9P-1), GS-168AT2, and developed a new monoclonal antibody directed towards the latter. We measured the expression of CD9 and CD9P-1 in human lung tumours (hLTs), and monitored the level of CD9 in NCI-H460, *in vitro* and *in vivo*, in the presence and absence of GS-168AT2.

**Results:**

Loss of CD9 is inversely related to the expression of CD9P-1, which correlates with the metastatic status of hLT (*n*=55). *In vitro*, GS-168AT2 is rapidly internalised and degraded at both the membrane and cytoplasm of NCI-H460, and this correlates with the association of GS-168AT2 with both CD9 and CD81. Intraperitoneal injections of GS-168AT2 in NCI-H460-xenografted *Nude* mice led to drastic inhibition of tumour growth, as well as to the downregulation of CD9, but not of CD81, in the tumour core.

**Conclusion:**

These findings show for the first time that CD9P-1 expression positively correlates with the metastatic status of hLT, and that the upregulation of CD9P-1 expression could be one of the mechanisms underlying the loss of CD9 in solid tumours. Our study also reveals that, under certain conditions, loss of CD9 could be a tumour growth-limiting phenomenon rather than a tumour growth-promoting one.

The tetraspanin protein family consists of 33 similar but distinct transmembrane proteins characterised by four transmembrane domains, two intracellular tails corresponding to the N- and C-termini of the protein, small and large extracellular spans, and some conserved amino-acid structures within both small and large extracellular domains. They are, nonetheless, structurally distinct, as they show 50–70% of sequence variability (for review see Charrin *et al* (2009)). Functionally, it is well established that tetraspanins associate with each other and with other cell surface proteins and receptors to form functional signalling platforms (Maecker *et al*, 1997; Boucheix *et al*, 2001; Boucheix and Rubinstein, 2001; Hemler, 2003, 2005; Tarrant *et al*, 2003; Yunta and Lazo, 2003; Lazo, 2007).

CD9, the most studied tetraspanin, is known to function in multiple cell events, including membrane fusion (Chen *et al*, 1999), differentiation (Xu *et al*, 1994; Choi *et al*, 2008), and cell motility (Kotha *et al*, 2008; Takeda *et al*, 2008), and seems to have a key role in metastasis (Zöller, 2009). Clinical observations suggest that downregulation of CD9 is associated with the progression of solid tumours. Recently, it was shown that small-cell lung cancer cell lines have no or very few CD9 (Funakoshi *et al*, 2003). Furthermore, loss of CD9 has been correlated with a higher motility and metastatic potential of the following tumour cells: lung (Higashiyama *et al*, 1995; Funakoshi *et al*, 2003; Wang *et al*, 2005), oral (Kusukawa *et al*, 2001), oesophageal (Uchida *et al*, 1999), ovarian (Houle *et al*, 2002), cervical (Sauer *et al*, 2003), and gastric (Murayama *et al*, 2002). Although CD9 has been investigated in the progression and invasion of tumours, nothing or little has been shown about its main partner, an Ig superfamily member called partner 1 of CD9 (CD9P-1)/FPRP/EWI-F.

Partner 1 of CD9 is a glycosylated, type 1 integral membrane protein (André *et al*, 2007), originally identified and characterised because of its ability to associate with and inhibit [^3^H]prostaglandin F2*α* binding to its receptor (Orlicky, 1996). Partner 1 of CD9 is associated with lipid accumulation (Orlicky *et al*, 1998), and gangliosides are a regulatory component of tetraspanin complexes and in particular of CD9 (Ono *et al*, 2001). Partner 1 of CD9 has been identified as a strong partner of protein CD9 (Charrin *et al*, 2001; Stipp *et al*, 2001) and as a member of the tetraspanin web (Charrin *et al*, 2003). Interestingly, we have shown that TNF*α* induces CD9P-1 expression in human endothelial cells (hEC), and that a truncated form of CD9P-1, named GS-168AT2, corresponding to the most membrane-adjacent part of the integral protein, dose-dependently inhibited *in vitro* angiogenesis (Colin *et al*, submitted).

In this study, we investigated the gene expression of CD9P-1 in human lung tumour (hLT) samples, and show that CD9P-1 expression at both transcriptional and translational levels correlates with the metastatic status of lung tumours, in particular at the migratory edge of the tumours. Incubation of GS-168AT2 with the hLT cell line NCI-H460 leads to the association of GS-168AT2 with both CD9 and CD81, and treatment of NCI-H460-xenografted *Nude* mice with GS-168AT2 leads to a drastic inhibition of tumour growth, which is associated with the *in vivo* downregulation of CD9 in tumours.

## Materials and methods

### Animals, cell lines, and products

Female BALB/C (8 weeks) and BALB/C nu/nu (5 weeks) were purchased from Charles Rivers (St Germain, France). Myeloma cell lines Sp2/O-Ag14, PEG, and HAT were obtained from Sigma (St-Quentin-Fallavier, France), whereas the NCI-H460 cell line was obtained from American Type Culture Collection. Phosphate-buffered saline, Trypsin-EDTA (Versene, Lonza, Levallois-Perret, France), fetal calf serum (FCS), and culture medium were from Eurobio (Courtaboeuf, France). Superscript II enzyme and RNase inhibitor were from Invitrogen (Cergy Pontoise, France) and the *Taq* polymerase enzyme was from New England Biolabs (Wilburg, UK). CD9 polyclonal antibodies (Clone H-110), CD81 monoclonal antibody (clone 5A6, sc23962), anti-mouse-HRP or anti-rabbit-HRP were from Santa Cruz. The 1F11 mAb was kindly provided by Dr E Rubinstein (U602, INSERM, Villejuif, France)

### Cell culture

The NCI-H460 cell line was grown in RPMI containing 10% FCS at 37°C and 5% CO_2_ humidified atmosphere. The absence of mycoplasms was confirmed by using the PCR Mycoplasma Detection kit (Takara, Lonza).

### hLT biopsies and RT–PCR

Biopsy samples were collected from patients with pulmonary tumours by curative resectional surgery and were kindly given by the Institut Mutualiste Montsouris (Paris, France) with the agreement of its ethical committee. Tumour staging was based on the pTNM pathological classification. The 55 hLTs were classified into four groups on the basis of the pTNM pathological classification, where T (*cis* 1–4) represents the size or direct extent of the primary tumour size; N represents the degree of regional lymph node metastasis (being: N0, tumour cells absent from regional lymph nodes; N1, closest or small number of regional lymph node metastasis present; N2, tumour spread to a number of and relatively distant regional lymph nodes; N3, tumour spread to more distant or numerous regional lymph nodes); and M represents metastasis to distant organs (beyond regional lymph nodes). Thus, throughout our study, we considered pT × N0 as being the non-metastatic primary hLT, pT × N1 as the weakly metastatic primary hLT, pT × N2 as the highly metastatic primary hLT, and pT × M as the highly metastatic secondary hLT (tumours from distant organs that metastased into the lungs).

Tumours and their peripheral tissues recognised as healthy tissues were immediately immersed into RNA conservative solution and conserved at −80°C. For semiquantitative RT–PCR, total RNA was extracted using a NucleoSpin RNA II kit (Macherey Nagel, Lonza). One microgram of total RNA was reverse-transcribed using Superscript III reverse transcriptase according to the manufacturer's instructions. The generated cDNAs were amplified with *Taq* polymerase according to the manufacturer's instructions using primers for CD9P-1 (5′-AGGTCCACTGCAGGGGGTTA-3′ and 5′-TTCCCCTTTGGAAGAGAGAGCA-3′); for CD9 (5′-TTGCTGTCCTTGCCATTGGA-3′ and 5′-CACTGGGACTCCTGCACAGC-3′); for GAPDH (used as the internal control) (5′-AGCTCACTGGCATGGCCTTC-3′ and 5′-GAGGTCCACCACCCTGTTGC-3′). The reaction mixtures were subjected to 30 PCR amplification cycles (30 s at 94°C, 30 s at 60°C, 1 min at 72°C). The amplified DNA samples resolved on 1.5% agarose gels, were visualised with ethidium bromide, and quantified with Gene tools software (Syngene, Lonza). For each biopsy including tumour core and peripheral tissues, CD9 and CD9P-1 expression levels were quantified relative to their respective GAPDH, and results were expressed as the mean±s.d. of three independent experiments.

### Cloning, production, and purification of the recombinant protein GS-168AT2

The truncated form of CD9P-1, GS-168AT2, was cloned, produced, and purified according to Colin *et al* (Submitted). We have also cloned, produced, and purified another recombinant protein corresponding to a truncated form of the cell surface tetraspanin 7/TM4SF2 (gene accession number: emb∣CAB65594.1∣; gene ID: 7102 TSPAN7) (amino acid no. 176–218). The purified recombinant protein has a molecular mass of 16 kDa and is used as a negative control protein (NCP) in animal experiments.

### Immunisation of animals and generation and selection of hybridomas

Female BALB/C mice (8 weeks) were intraperitoneally (i.p.) injected with 100-*μ*g GS-168AT2 supplemented with Freund's complete adjuvant (1/1 (v/v)), followed by two injections at 3-week intervals, and a final boost with 100-*μ*g GS-168AT2. After 3 days, the spleen was harvested and fused with the myeloma cell line Sp2/O-Ag14 using 50% PEG diluted in RPMI-1640 medium, and the fused cells were cultured in RPMI-1640 containing 15% FCS and HAT (Eurobio) and 10% hyridoma cloning factor (PAA Laboratories, Les Mureaux, France). Supernatants were screened by ELISA using GS-168AT2 as antigen, His-Tag proteins (developed in our laboratory) as control, and the anti-mouse IgG-HRP conjugate. Positive hybridomas were isolated, subjected to three rounds of dilution limit, and one hybridoma was selected. This was cloned, and by extension the antibody secreted was called 229T mAb.

### Ascitic fluids production and mAbs purification

BALB/c mice were i.p. injected with 300 *μ*l of incomplete Freund's adjuvant, inoculated with 6 × 10^6^ hybridoma cells 24 h later, and ascitic fluid was collected 8 days later. Ascitic fluids were diluted in 20 mM PBS and loaded onto a G-protein affinity column (Hitrap protein; Amersham, Pantin, France) mounted onto an HPLC system (Actabasic; Amersham). Following extensive washing, the IgG fractions were pulled together by elution with 0.1 M glycine (pH 2.7). Proteins were determined by Bradford assay, purity was controlled by SDS–PAGE, and titration was realised by ELISA as described above.

### Immunoprecipitation

The cell monolayer was washed twice with cold PBS, and lysed with 1% Triton X-100 buffer for 1 h at 4°C. Proteins were immunoprecipitated by adding 2 *μ*g of 229T mAb or 5 *μ*g of anti-CD9 or anti-CD81 (1 h at 4°C); the immune complexes were harvested with protein G-Sepharose beads (Santa Cruz), washed with lysis buffer, resolved in SDS–PAGE, and proteins were transferred to PVDF membrane and revealed with the indicated antibody.

For immunoprecipitation in denaturing conditions, cell layers were washed three times with cold PBS, and lysed in cold lysis buffer 1% SDS, 5 mM EDTA, and 10 mM DTT (denaturing conditions). To demonstrate the ability of 229T mAb to immunoprecipitate GS-168AT2 and CD9P-1 proteins, H-460 cell lysates treated in denaturing or non-denaturing conditions were supplemented with either GS-168AT2 (1 *μ*g) or vehicle.

### Cell membrane preparations

Cytosolic and nuclear extracts were prepared by the modified method of Dignam *et al* (1983). After different incubation times with GS-168AT2, NCI-H460 cells were washed with cold PBS, lysed in ice-cold lysis buffer (5 mM HEPES, 1.5 mM MgCl_2_, 10 mM KCl, 0.5 mM dithiothreitol, 0.1 mM PMSF, and 0.1 mM aprotinin), and centrifuged at 10^4^ **g** for 30 min at 4°C. The supernatant was collected as the cytosolic fraction. Pellets were homogenised in the above-mentioned lysis buffer containing 2% Triton X-114 (Sigma) and centrifuged at 800 **g** for 10 min at 4°C. The supernatant was collected and is referred to here as the membrane fraction.

### Immunohistochemistry on tumour sections

After de-paraffination with Histolemon reagent (CarloErba, Lonza) and rehydration, epitopes were retrieved with MS-Unmarker (Diapath) using a microwave oven. Endogenous peroxidases were blocked by 0.3% H_2_O_2_ for 30 min. After washing with PBS, sections were blocked with horse serum (1 : 100) for 30 min, and incubated in a sodium phosphate and citric acid buffer (pH=8.4). Ac 229 (100 *μ*g ml^−1^) was incubated for 30 min at 37°C. Bound antibodies were detected with biotinylated horse anti-mouse Ig and avidin-HRP (Cliniscience, Montrouge, France). Counterstaining was performed with hemalun Mayer, followed by treatment with a bluing reagent.

### Tumour xenografts in nude mice and GS-168AT2 administration

All experiments were reviewed by Genopole's institutional animal care and use committee, and performed in accordance with institutional guidelines for animal care. Female BALB/c nu/nu mice (*n*=31) were used at 5–6 weeks of age. The animals were housed in laminar air-flow cabinets under pathogen-free conditions with a 14 h light/10 h dark schedule, and fed autoclaved standard chow and water *ad libitum*. NCI-H460 cells (5 × 10^6^ cells in 200 *μ*l of serum-free RPMI) were subcutaneously injected into the right flank of mice. After engraftment (10 days), tumour volume (TV) was measured (Balsari *et al*, 2004) and animals were randomised and separated into seven groups of five animals each, to be treated by i.p. injection (200 *μ*l per injection) every other day for 16 days (eight injections). Control mice (group 1) received the vehicle (2 M urea in 0.9% saline). *Cis*-diammine platinum II dichloride (CDDP; Sigma) was dissolved in 0.9% saline at 0.5 mg ml^−1^ and injected at a dose of 5 mg kg^−1^ (group 2).

NCP was dissolved in vehicle at 1.5 mg ml^−1^ and injected at a dose of 15 mg kg^−1^ (group 3). GS-168AT2 was dissolved in vehicle at 1.5 mg ml^−1^ and injected at a dose of 15 mg kg^−1^ (group 4). In group 5, mice received both CDDP and GS-168AT2. Tumour volume and body weight were measured every other day over the treatment period (16 days).

In another series of experiments, two groups of three animals each were xenografted with NCI-H460 as above, after which, starting from day 10 post xenograft, animals were treated daily with either vehicle or GS-168AT2 for 5 days, and tumour blocks were harvested at the end of treatment for analysis.

### Statistics

All data were analysed with Prism 5 using the Mann–Whitney test and the unpaired *t*-test. For CD9 and CD9P-1 expression, medians were calculated and considered significantly different when the two-tailed *P*-value was <0.05. All data are presented as mean±s.d., where *n* is the number of independent experiments. A *P*-value <0.05 was considered to be significant.

##  Results

### Production and characterisation of the new mAb antitruncated form of CD9P-1

Following immunisation, selection, and limit dilution, isotyping results show that the culture medium of the selected clone (clone number 229T) contained only IgG1. This IgG1 recognised GS-168AT2 in ELISA, and failed to recognise CD9P-1 in flow cytometry (data not shown).

**Figure 1:**
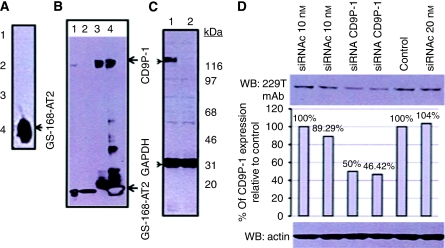
Characterisation of 229T mAbs. (**A**) GS-168AT2 (4) and other His-Tag recombinant proteins (2, 3) or vehicle (1) were immobilised on nitrocellulose membrane, immunoblotted with 229T mAb, and revealed with anti-mouse-HRP antibody. (**B**) Cell lysates of NCI-H460 tumour supplemented with GS-168AT2 (lane 1) or GS-168AT2 alone (lane 2), as well as immunoprecipitates obtained with 1F11 mAb either from NCI-H460 tumour lysates alone (lane 3) or from those supplemented with GS-168AT2 (lane 4) were resolved in SDS–PAGE and immunoblotted with 229T mAb. Representative image of the western blot of three independent experiments is presented. (**C**) Cell lysates of either wild human microvascular endothelial cells (lane 1) or human microvascular endothelial cells constitutively expressing antisense transcript of CD9P-1 (lane 2) were resolved in SDS–PAGE and immunoblotted with 229T mAb. Representative image of the western blot of four independent experiments is presented. (**D**) NCI-H-460 cells were transfected either with nonspecific siRNA (siRNAc) as control or with siRNA specific to CD9P-1 (siRNA CD9P-1), or incubated with siRNA-free transfection cocktail (control). Cell lysates were prepared and resolved in SDS–PAGE and immunoblotted either with 229T mAb (upper panel) or with anti-actin antibody (lower panel) as internal control. Partner 1 of CD9 expression was then appreciated (middle panel) relative to the internal control (actin). Results representative of two independent experiments are presented.

Experimentation with 229T mAb showed that, in dot-blot assays, it did not recognise many His-Tag proteins; however, it recognised GS-168AT2 (Figure 1A). The 229T mAb did not immunoprecipitate CD9P-1 under native conditions, but it did under denaturating conditions (data not shown). In western blot, 229T mAbs recognised CD9P-1 in cell lysates and in GS-168AT2 alone (Figure 1B, lanes 1 and 2, respectively). To further validate the 229T mAb, we used 1F11 mAb (Charrin *et al*, 2001) to immunoprecipitate CD9P-1 from cell lysates alone or supplemented with GS-168AT2. Resolving the immunoprecipitates in SDS–PAGE and western blotting showed that 229T mAb recognised the band of 135 kDa immunoprecipitated with 1F11 mAb, which corresponds to CD9P-1 (Figure 1B, lanes 3 and 4), as well as 18 and 36 kDa bands corresponding to the monomer and dimer of GS-168AT2, respectively (Figure 1B, lane 4). Of interest, the 229T mAb also recognised other bands of molecular weight lower than that of CD9P-1 (Figure 1B, lanes 3 and 4), which could be proteolytic fragments of CD9P-1 generated either from the preparation of cell lysates or from the natural turnover of CD9P-1.

Human microvascular endothelial cells (HMECs) were also transformed with a pci-neovector coding for an antisense transcript specific to CD9P-1. Western blotting of cell lysates of wild HMEC and those of the HMEC cell line stably expressing pci-neovector coding for an antisense transcript specific to CD9P-1 showed that the 229T mAb recognised the band (135 kDa) corresponding to CD9P-1 in lysates of wild HMEC (Figure 1C, lane 1), but not in lysates of the HMEC cell line stably expressing pci-neovector coding for an antisense transcript specific to CD9P-1 (Figure 1C, lane 2).

Finally, the NCI-H460 tumour cell line was transfected either with siRNA specific to CD9P-1 or with nonspecific siRNA as control. Immunoblotting of cell lysates showed that the 229T mAb recognised the band of CD9P-1 (≈135 kDa) to a much less extent (≈50% decrease) in lysates issued from cells transfected with siRNA specific to CD9P-1 compared with those issued from cells transfected with nonspecific siRNA (Figure 1D).

### CD9P-1 is overexpressed in metastasis

**Figure 2:**
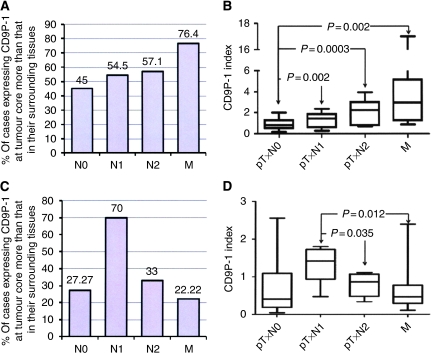
Partner 1 of CD9 expression correlates with the metastatic status of human lung tumours. (**A**) Fifty-five biopsy samples of hLTs, as well as of their surrounding tissues, were classified according to the TNM staging system, and the expression of CD9P-1 was quantified at both the tumour core and the surrounding tissues by RT–PCR relative to GAPDH. Results are presented as the percentage of cases (for each class of tumours) that have a ratio >1 of CD9P-1 at the tumour core relative to CD9P-1 at the surrounding tissue. (**B**) Distribution of the CD9P-1 index (ratio of CD9P-1 at the tumour core relative to CD9P-1 at surrounding tissues) of the different classified groups. (**C**) The expression of CD9 was quantified at both the tumour core and surrounding tissues by RT–PCR relative to GAPDH using hLT as in (**A**). Results are presented as the percentage of cases (for each class of tumours) that have a ratio >1 of CD9 at the tumour core relative to CD9 at surrounding tissues. (**D**) Distribution of the CD9 index (ratio of CD9 at the tumour core relative to CD9 at surrounding tissues) of the different classified groups.

We studied 55 hLT biopsies (including 17 cases of secondary hLT) for CD9 and CD9P-1 gene expression, using the CD9 index (CD9 mRNA level in tumour core relative to CD9 mRNA in surrounding tissues) and the CD9P-1 index (CD9P-1 mRNA level in tumour core relative to CD9P-1 mRNA in surrounding tissues). Nine of 20 cases (45%) of primary hLT (pT × N0) showed CD9P-1 index superior to 1, indicating more CD9P-1 mRNA within tumour cores than in their surrounding tissues. In all, 54% (6 of 11) cases of metastatic primary hLT pT × N1 and 57.1% (3 of 7) cases of pT × N2 have CD9P-1 superior to 1. For secondary hLT, 76.4% (13 of 17 cases) expressed CD9P-1 index superior to 1 (Figure 2A).

Intergroup analysis of the magnitude of CD9P-1 index and its distribution showed that there was no significant difference between the groups of primary hLT (pT × N0) and metastatic primary hLT pT × N1 (mean CD9P-1 index±s.e.m.: 0.872±0.105 and 1.295±0.224, respectively; *P*=0.0619; 95% CI=−0.868 to 0.0224; Figure 2B). In contrast, the group of metastatic primary hLT (pT × N2) showed a highly significant difference (mean CD9P-1 index±s.e.m.: 2.179±0.436; *P*=0.0003; 95% CI=−1.938 to −0.6739) compared with the group of primary hLT (pT × N0) (Figure 2B).

The secondary lung metastasis group hLT (M) also showed a significant difference (mean CD9P-1 index±s.e.m.: 4.098±1.041; *P*=0.002; 95% CI=−5.184 to −1.266) compared with the group of primary hLT (pT × N0) (Figure 2B).

For CD9 gene expression, 27.3% of non-metastatic primary lung tumours (pT × N0) expressed CD9 mRNA more at tumour cores than in their surrounding tissues (Figure 2C). CD9 gene expression within metastatic primary lung tumours (pT × N1and pT × N2) and secondary lung metastasis (M) showed a direct correlation between the downregulation of CD9 expression and the metastatic status of hLT; 70, 30, and 22.2% of pT × N1, pT × N2, and M, respectively, expressed CD9 more in tumour cores than in their surrounding tissues (Figure 2C).

The magnitude and distribution analysis of CD9 showed that there were no significant differences (*P*>0.05) between the group of primary hLT (pT × N0) (mean CD9 index±s.e.m.: 0.807±0.269) and any of the other groups, namely, pT × N1 (mean CD9 index±s.e.m.: 1.31±0.138), pT × N2 (mean CD9 index±s.e.m.: 0.79±0.14), and M (mean CD9 index±s.e.m.: 0.663±0.118). In contrast, the group of metastatic primary hLT (pT × N2) showed a significant difference (*P*=0.035; 95% CI=0.0428–0.999) compared with the group of metastatic primary hLT (pT × N1) (Figure 2D). The secondary metastasis group hLT (M) also showed a highly significant difference (*P*=0.002; 95% CI=0.262–1.041) compared with the group of primary hLT (pT × N0) (Figure 2D).

**Figure 3:**
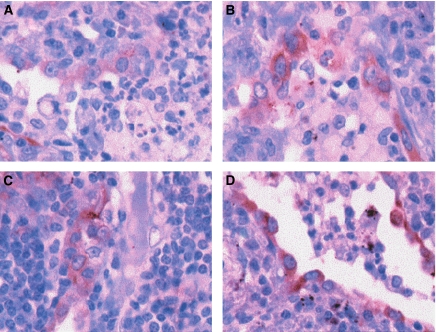
Partner 1 of CD9 protein expression is associated with the edge migratory front of tumours. CD9P-1 immunolabelling with 229T mAb in pulmonary metastasis adenocarcinoma from the prostate (**A**–**C**). The strong labelling with the 229T mAb of the edge migratory front of tumours is shown in (**D**).

Partner 1 of CD9 expression at the protein level was also investigated. Immunostaining of human cancer biopsy samples with 229T mAb showed that there was important staining of individual tumour cells (migratory cells) within tissue sections (Figures 3A–C), suggesting that an induced expression of CD9P-1 is associated with the migratory character of tumour cells. The possibility that the upregulation CD9P-1 protein expression associates with the migratory character of tumour cells was also more visible in Figure 3D, in which tumour cells at the migratory edge were clearly positively stained.

### Tumour cell interaction with GS-168AT2 leads to its degradation

**Figure 4:**
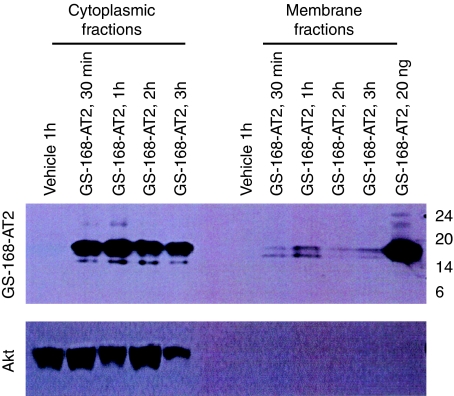
GS-168AT2 interaction with tumour cell led to its internalisation and degradation. The NCI-H460 layer was incubated with GS-168AT2 for the indicated time, washed, lysed, and fractionated into cytosol and membrane fractions. The two fractions were resolved in SDS–PAGE, followed by immunoblotting with 229T mAb. The two fractions were also resolved in SDS–PAGE, followed by immunoblotting with mAb anti-protein kinase B (Akt) (a cytoplasmic protein); results showed that Akt was only detectable in the fraction of cytosol and not in the membrane fraction, suggesting that there were no intercontaminations between the two fractions. Representative image of two independent experiments is presented.

To provide insight into the fate of GS-168AT2, cells incubated with it were fractionated into cytoplasm and membrane fractions. Western blot with 229T mAb showed weak quantity with a peak of intact GS-168AT2 at 1 h associated with a degraded form (about 16 kDa) of GS-168AT2 throughout the kinetics at the membrane fractions, indicating that GS-168AT2 could be cleaved at membranes as early as 30 min (Figure 4). Results of the cytoplasm fractions showed that there is an important quantity of intact GS-168AT2 throughout the kinetics associated with increasing quantities of a degraded form (about 15.5 kDa) of GS-168AT2 up to 2 h, suggesting that GS-168AT2 interacts with the NCI-H460 surface, leading to its degradation (Figure 4).

### GS-168AT2 interacts with CD9 and CD81

**Figure 5:**
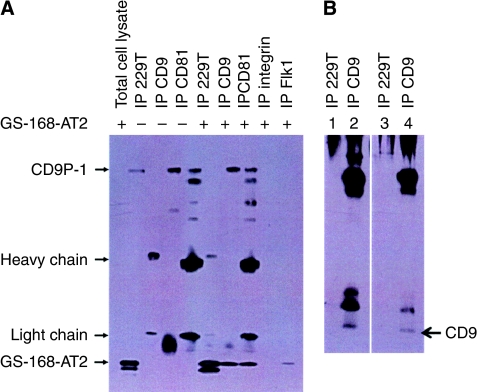
The degradation of GS-168AT2 is associated with its interaction with CD9 and CD81. (**A**) NCI-H460 cells were incubated for 2 h with (lanes labelled with +) or without (lanes labelled with −) GS-168AT2, followed by lysing with native lysis buffer. Proteins were immunoprecipitated with 229T mAb (lanes labelled with IP 229T mAb), anti-CD9 antibody (lanes labelled with IP CD9), anti-CD81 antibody (lanes labelled with IP CD81), anti-*β*1-integrin antibody (lanes labelled with IP integrin), or anti-vascular endothelial factor receptor (IP Flk 1) (lanes labelled with IP Flk1). The obtained immunoprecipitates or total cell lysates of cells incubated with GS-168AT2 (lane labelled with total cell lysate) (as control) were resolved in SDS–PAGE under reducing conditions and western blotted with 229T mAb. GS-168AT2 was undetectable with the immunoprecipitates obtained with the anti-*β*1-integrin (IP *β*1-integrin) antibody and with the antivascular endothelial cell growth factor receptor (IP Flk 1), but was detectable with the immunoprecipitates obtained with 229T mAb (IP 229T mAb). Representative image of the western blot of three independent experiments is presented. (**B**) The immunoprecipitates obtained with either 229T mAb (lanes labelled with IP 229T mAb) or anti-CD9 antibody (lanes labelled with IP CD9), in the presence or absence of GS-168AT2 as in panel A of this figure, were also resolved in SDS–PAGE and immunoblotted with anti-CD9 antibody. CD9 was well immunoprecipitated with anti-CD9 antibody under our experimental conditions, as described in panel A.

The possible interaction of GS-168AT2 with CD9 and CD81 was investigated. NCI-H460 cells were incubated for 2 h with GS-168AT2, lysed under non-denaturing conditions, and immunoprecipitation was realised with either anti-CD9, anti-CD81, or 229T mAb. As described above, 229T mAb did not immunoprecipitate CD9P-1 under non-denaturing conditions, but led to the immunoprecipitation of GS-168AT2 and fragments, confirming the results observed by western blot. Immunoprecipitation with 229T mAb did not co-precipitate CD9, probably because of the engagement of the epitope of GS-168AT2 with CD9. On the other hand, immunoprecipitation of either CD9 or CD81 led not only to the co-precipitation of CD9P-1 but also to the co-precipitation of GS-168AT2, suggesting the direct interaction between GS-168aT2 with CD9 and CD81 (Figure 5A). Traces of cleaved fragments were also observed.

To ensure that CD9P-1 did not precipitate with other cell surface proteins, we have also immunoprecipitated integrin-*β*1 and the receptor of vascular endothelial cell receptor (Flk). Immunoblotting with 229T mAb revealed that there was no detectable co-precipitation of GS-168AT2 with these cell surface proteins (Figure 5A), thereby confirming that GS-168AT2 specifically co-precipitated with CD9 and CD81.

The immunoprecipitates obtained with 229T mAb or anti-CD9 were also resolved in SDS–PAGE and blotted with anti-CD9. Results showed that CD9 was effectively immunoprecipitated (Figure 5B).

### GS-168AT2 inhibits the *in vivo* hLT growth

**Figure 6:**
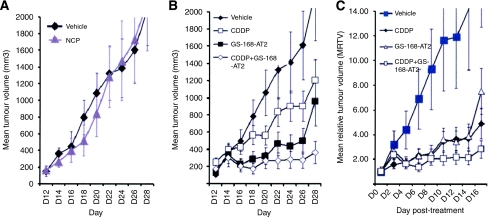
GS-168AT2 inhibits *in vivo* tumour growth. (**A**) MTV curve of mice bearing NCI-H460 tumours and treated with vehicle or nonspecific protein (NCP) at 5 mg /kg^−1^. (**B**) MTV curve of mice bearing NCI-H460 tumours and treated with vehicle, CDDP at 5 mg kg^−1^, GS 168A-T2 at 15.0 mg kg^−1^, or combined GS 168A-T2 at 15.0 mg kg^−1^ and CDDP at 5 mg kg^−1^. (**C**) RMTV evolution of the same groups as in (**B**).

**Figure 7:**
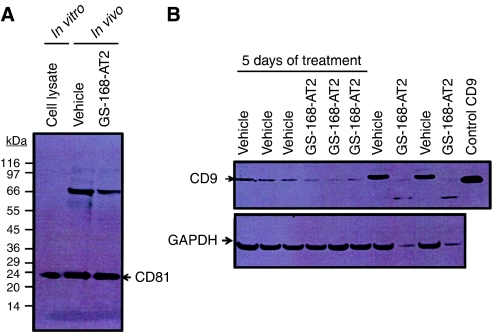
GS-168AT2 inhibits the *in vivo* tumour growth associated with drastic *in vivo* downregulation of CD9 in the tumour core, but not in CD81. (**A**) Tumour masses were extracted from animal cores at the end of treatment, homogenised, resolved in SDS–PAGE, and immunoblotted with anti-CD81 antibody. Commercial control cell lysates for CD9 (Santa Cruz) were used as control. Lane labelled with vehicle: tumours from animals treated with vehicle; lane labelled with GS-168AT2: tumours from animals treated with GS-168AT2. (**B**) Tumour masses were extracted from animals at the end of treatments (either after five successive days of treatment, lanes labelled under 5 days of treatment, or after complete treatment, as described in Figure 6 and under Materials and Methods section), homogenised, resolved in SDS–PAGE, and immunoblotted with either anti-CD9 antibody (upper panel) or anti-GAPDH antibody (lower panel) as control. CD9 expression was then appreciated (middle panel) relative to the internal control (GAPDH). Results representative of two independent experiments are presented.

All nude mice bearing NCI-H460 tumours survived during the therapy. Before therapy, there were no significant differences among nude mice with respect to weight and TV. The results of mean tumour volume (MTV) and mean relative tumour volume (MRTV) are shown in Figures 6A–C. The MTV at day 28 in mice of group 2 treated with NCP is similar to that of mice treated with vehicle (group 1) and there were no significant differences between the two groups (*P*>0.05) (Figure 6A). The MTV at day 28 in mice of group 3 treated with CDDP (1203.48±240.69 mm^3^) is significantly (*P*=0.001) less than that of mice of vehicle group 1 (2220.34±555.08 mm^3^) (Figure 7B). The MTV at day 28 was decreased (*P*=0.001) in mice of group 4 treated with GS-168AT2 alone (958.38±287.51 mm^3^). The first statistical significance between group 1 and the other groups was observed at day 4 post treatment for groups 4 and 5 (Figure 6B). An important reduction in MTV (364.22±105.62 mm^3^) was observed in animals from group 5 compared with those from group 1 (*P*=0.0001).

These results were confirmed by analysis of MRTV at day 28 (Figure 6C). GS-168AT2, when used as a monotherapy, reduced tumour growth by at least 50% compared with group 1. When combined with CDDP, however, this reduction reached 83%.

Tumours were extracted from animals at the end of the treatments and lysed. Immunoblotting with the anti-CD81 antibody of these lysates showed that tumours treated with vehicle and those treated with GS-168AT2 contained equivalent amounts of CD81 at the end of the treatments (Figure 7A).

Immunoblotting with the anti-CD9 antibody of tumour lysates at the end of the treatments showed that tumours of vehicle-treated animals contained an important quantity of CD9. However, in tumours of GS-168AT2-treated animals, only trace amounts of CD9 were detected (Figure 7B). To give further insight into the chronology of the loss of CD9 with treatment with GS-168AT2, two groups of animals (three animals per group) xenografted with NCI-H460 tumours as above were treated for 5 days with either vehicle or GS-168AT2, under the same conditions as above. Analysis of CD9 showed that tumours from animals treated with GS-168AT2 for five successive days have less CD9 than those from animals treated with GS-168AT2 for 5 days (Figure 7B).

## Discussion

In this study, we have shown that CD9P-1 was upregulated in human cancer metastasis, suggesting that this could be one of the mechanisms underlying the loss of CD9 expression in solid tumours. Our study also shows that this loss of CD9, when associated with the increase in CD9P-1 expression, could be a tumour growth-limiting phenomenon. These findings are supported by several direct facts, including the following: (i) CD9P-1 expression correlated with the metastatic status of hLT and was inversely proportional to CD9 expression; (ii) the truncated form of CD9P-1, GS-168AT2, was rapidly internalised by tumour cells correlating with its interaction with both CD9 and CD81; and most importantly, (iii) treatments of hLT-xenografted *Nude* mice with GS-168AT2 led to a drastic inhibition of hLT growth that was associated with an important *in vivo* downregulation of CD9 in the tumour core, but not in CD81.

Loss of CD9 is correlated with cancer metastasis in many solid tumours (Higashiyama *et al*, 1995; Funakoshi *et al*, 2003; Wang *et al*, 2005). Although CD9P-1 is present in a large panel of cancer cell lines (Charrin *et al*, 2001; Vidal-Laliena *et al*, 2005), the role of CD9P-1 in oncogenesis and metastasis remains unknown. Our results show that CD9P-1 expression strongly correlates with the metastatic status of hLT, as 45% of primary non-metastatic hLTs express CD9P-1 more than their surrounding tissues, whereas 55% of metastatic primary hLTs and 83% of secondary hLT metastases express CD9P-1 more than their surrounding tissues. It was shown recently that the transfection of HEK-293 cells with CD9P-1 coding vector increased cell migration (Chambrion and Le Naour, 2010). Our results extend these *in vitro* results, and showed that the expression of CD9P-1 localises at the migratory edge of human tumours.

Interestingly, CD9 expression was inversely proportional to both CD9P-1 expression and the metastatic status of hLT. It is worth mentioning, however, that although the association of the loss of CD9 with the metastatic status of tumours is highly significant and in agreement with the literature (Higashiyama *et al*, 1995; Adachi *et al*, 1998), our appreciation of CD9 expression within the tumour core was certainly overestimated. Indeed, it is widely established that tumour angiogenesis correlates with the metastatic status of tumours, indicating that higher the levels of hEC (neovessels) that are present within the tumour core, more important the metastatic potential of the tumour. Recently, it was shown that CD9 is highly expressed in hEC (Klein-Soyer *et al*, 2000), suggesting that an important source of CD9 that was detected within the tumour core could originate from the invading neovessels.

We previously reported that GS-168AT2 has potent antiangiogenic activity correlating with both, its degradation by hEC and CD9 downregulation (Colin *et al*, Submitted). In this study, we show that CD9 downregulation *in vivo* is associated with the degradation of GS-168AT2. Our data show that most of the degraded form was present in the cytosolic fraction, suggesting that GS-168AT2 was internalised by NCI-H460, but we cannot exclude the contamination of cytosolic fractions by membrane-bound GS-168AT2. The degradation of GS-168AT2 by NCI-H460 cells is associated with CD9 downregulation *in vivo*, suggesting that either native GS-168AT2 or the GS-168AT2-derived fragments directly or indirectly interact with CD9, leading to the internalisation of the complex. These results are consistent with earlier results showing that CD9P-1 is a partner of CD9 (Charrin *et al*, 2001).

Treatment of NCI-H460-xenografted mice with GS-168AT2 (15 mg kg^−1^) alone for 16 days reduced tumour growth by about 50%. This effect was similar to that of treatment with CDDP alone (5 mg kg^−1^), and the combined treatment of GS-168AT2 and CDDP led to over 83% inhibition of tumour growth. This illustrates the greater efficacy of bitherapies (chemotherapy associated with antiangiogenic therapy) at reducing hLT proliferation. The antiangiogenic activity of GS-168AT2 most probably accounts for most of the antitumour growth effect (Colin *et al*, Submitted). The efficacy of GS-168AT2 could be the sum of its direct effect on tumour cells and its antiangiogenic activity.

In conclusion, pharmacological modulation of the tetraspanin web affects multiple mechanisms, including tumour growth, and angiogenesis could constitute an efficient target for cancer therapies (Hemler, 2008).
